# Clinicians' Attitudes Toward Electronic Health Records in Saudi Arabia

**DOI:** 10.7759/cureus.56281

**Published:** 2024-03-16

**Authors:** Tourkiah Alessa

**Affiliations:** 1 Biomedical Technology Department, College of Applied Medical Sciences, King Saud University, Riyadh, SAU

**Keywords:** attitudes, clinicians, nurses, physicians, doctors, emrs, ehrs

## Abstract

Aim: This study explored physicians' and nurses’ attitudes toward an electronic health record (EHR) system and examined the features and factors that clinicians associated with the implementation of EHR systems.

Methods: A self-administered anonymous questionnaire with high reliability and validity was adopted from existing research to gather clinicians’ attitudes toward the EHR system implemented at King Khalid University Hospital, one of the biggest hospitals in Riyadh, Saudi Arabia.

Results: A total of 438 questionnaire responses were received from the participants; 240 of them were physicians and 198 were nurses. The participants had a mean age of 43.7 years (standard deviation (SD) 17.1), 213 (52.7%) were female and 207 (47.3%) were male. Most participants (424, 96.8%) had one or more years of experience using computers, and a majority (304, 69.4%) had one or more years of experience using EHR systems. Most physicians and nurses (214, 89.5% vs. 174, 87.9%) were satisfied with their hospital’s EHR system and felt that the system was highly usable and had the potential to improve communication between staff, facilitate easy storage of and access to information and lead to improved health outcomes for patients. The study found positive attitudes among clinicians concerning the quality of training and education around the new system (178, 74.2% of physicians vs. 142, 71.7% of nurses; p > 0.05) and toward leadership during the transition to HER (222, 92.5% vs. 183, 92.4%). On the other hand, a majority of nurses reported that the EHR system took longer to use and increased their workload compared with the previous analogue system (115 (47.9%) vs. 133 (67.2%); p ≤ 0.01 and 46.7% vs. 112 (64.1%)). A large majority of physicians and nurses surveyed (214 (89.2%) vs. 167 (84.3%)) stated that clinicians should be consulted in the design of such systems as a way to maximise the potential benefits of EHR and mitigate extra workload demands.

Conclusion: Most clinicians expressed overall satisfaction with the EHR system, but there were some areas of dissatisfaction among the respondents, such as increasing workload and stress among nurses. There is scope for further research to continue to explore physicians' and nurses’ attitudes toward EHRs and for future experimental studies that examine the impact of EHRs on clinician workloads, patient health outcomes and quality of care.

## Introduction

Electronic health record (EHR) systems are increasingly utilised in hospital settings, particularly in Saudi Arabia [1,2]. Their increasing popularity owes to their potential to reduce costs of healthcare provision, whilst improving patient outcomes and safety [3]. EHRs enable healthcare providers to offer better and safer care for their patients by enabling easier and more standardised access to the information medical professionals require [4].

In developed countries, such as the USA, the implementation of EHRs in primary care settings occurred relatively early and adoption rates are high (EHR systems were used in roughly 96% of private hospitals and 84% of general practices in 2017). In England, all patient records were digitised in 2015, and Australia undertook a similar computerisation initiative in 2016-2019 [5]. In developing countries, drives towards EHR implementation began later but have since gathered a significant pace. The Kingdom of Saudi Arabia is a leader in this field, given its relative financial prosperity and rapidly developing healthcare system. Saudi Arabia has made progress in implementing e-health in a range of care contexts, such as hospitals, laboratories and prescription services [6]. Prior research has examined the rate of uptake of these technologies, for instance using the Healthcare Information and Management Systems Society (HIMSS) adoption model with some care centres achieving a stage 7 accreditation, the highest stage possible [1,7]. EHR technologies used within KSA are sourced from a variety of developers, including Epic and Cerner, as well as other high-rated producers [8].

Globally, a significant body of research has examined stakeholders’ awareness of these EHR systems and begun to identify factors that influence their successful implementation, including the requirement for comprehensive training, and efficient and effective leadership in transitioning to these new systems [6,7]. Several research works have also identified dissatisfaction among physicians and nurses as a key factor affecting EHR adoption [8,9], which can be caused by financial issues; complexity of systems, including multiplicity of screens, options and navigational aids; and a lack of customizability. Jabali and Abdulla proposed that EHR systems should be regularly evaluated in terms of usability, perceptions and end-user satisfaction [7,10]. As clinicians (physicians and nurses) comprise the main users of EHR systems, their attitudes towards these systems play a role in influencing other medical professionals to accept their implementation and the advantages they bring. Their acceptance is therefore an essential consideration in EHR research. A better understanding of physicians’ and nurses’ acceptance has the potential to inform wider investigations into EHR use and improve implementation procedures [4].

Although some previous research has examined EHR use in Saudi Arabia and begun to assess Saudi physicians’ perceptions of these systems, there have been very few studies examining both nurses’ and physicians’ satisfaction [1,4,8,9] and relatively little research into the factors that influence clinicians’ attitudes towards EHRs and their implementation within this specific context [6,7]. 

The present study therefore addresses several previously unexamined issues: (1) It studies the implementation of EHR within one of Saudi Arabia’s largest teaching hospitals (700 beds), which is also a key referral centre for treatments and surgeries within KSA and which launched its EHR system in 2021, one year before the commencement of the current study. (2) It examines attitudes towards the use of this specific EHR system within KSA among different end users, including both physicians and nurses. The main objectives of this research are to 1) examine both physicians’ and nurses’ satisfaction with the adopted EHR system, 2) investigate their attitudes regarding the implementation of this system and 3) examine potential factors that might influence these perceptions, such as demographic details, training provision and so on. The results of this research can inform the hospital itself and provide guidance to facilitate successful transitions to such systems elsewhere.

## Materials and methods

A quantitative cross-sectional study, using a structured questionnaire, was conducted to collect accurate and anonymous data on participants’ satisfaction and perceptions of an EHR system [11].

Overview of the EHR system and setting

The EHR system used at the studied hospital is a commercially developed system, called Cerner PowerChart® (Oracle Cerner, USA), which is accessed via desktop computers and other devices (e.g., laptops) and launched in 2021, one year before the commencement of the study.

The study hospital is King Saud University Hospital, a tertiary-level hospital in Riyadh and one of the largest public teaching hospitals in Saudi Arabia, which houses 700 beds and a key referral centre for treatments and surgeries within KSA. It conducts an average of 14,231 procedures and delivers treatment to more than 1.2 million outpatients per year.

Participants

The target population was clinicians (physicians and nurses) using an EHR system in Riyadh, Saudi Arabia, comprising 30,000 doctors and nurses. A sample size of 398 was determined, considering a 95% confidence level, ±5% margin of error, 0.05 alpha error and 80% power level. These parameters guarantee precise and reliable findings, making the chosen sample size of 398 suitable for the study.

Due to the limited time and resources of this research, it was not practicable to gain access to the entire population. Therefore, a convenience sample was utilised to capture an accessible subset of the study population. Questionnaires were distributed to hospital physicians and nurses during morning meetings, with participants completing them after signing a consent form. The ethical approval was obtained from the hospital ethical committee (approval no. 22-369E).

Study tool

The study adopted the validated and reliable Clinical Information System Implementation Evaluation Scale (CISIES) questionnaire, which was developed by an expert panel based on a highly validated questionnaire [12] and tested to ensure its validity and enhance its content for evaluating users' satisfaction with EMR. The survey underwent rigorous testing and validation processes, demonstrating a high internal consistency of 0.94 Cronbach’s alpha, thus enhancing its reliability for assessing perceptions of EMR users [12,13] (see Appendix). The purpose of the questionnaire is to solicit clinicians’ perceptions of EHR [12]. It includes five questions regarding participant characteristics and 37 statements concerning EHR systems, based on a six-point Likert scale (“strongly disagree” to “strongly agree”).

The paper-based questionnaires, with information sheets attached, were distributed directly to hospital physicians and nurses during their morning meetings, and the participants were asked to complete the questionnaire after they signed a consent form. To protect anonymity, the participants were instructed to deposit their completed questionnaires in a box located at the reception of their respective departments, and this procedure was supervised by the hospital’s medical director. 

The questionnaire provides insights into individual aspects of clinicians’ perceptions of EHR. The participants’ satisfaction in response to each statement/question was characterised based on the Likert-point scale as either agree (“somewhat agree”, “agree” and “strongly agree”) or disagree (“somewhat disagree”, “disagree” and “strongly disagree”).

Data analysis

Once 438 completed questionnaires had been received, the data were subjected to descriptive and inferential analysis using IBM SPSS Statistics for Windows (IBM Corp., Armonk, New York, United States) to assess clinicians’ perceptions of EHR and factors surrounding the implementation of the EHR system. The software was used to provide the mean and percentage scores, among others. The two overall satisfaction items (“I overall prefer using the system than the old way of doing things” and “Overall, the introduction of the system has been effective”) were analysed jointly, and associations with demographic factors were investigated. The normality test was conducted before correlating overall satisfaction with demographic variables (e.g. age), and the Shapiro-Wilk test results yielded p < 0.01, confirming that the variables were not normally distributed. The correlation was done using Spearman’s test, which is appropriate to examine potential links between overall satisfaction and demographic variables of age, experience of EHR, experience using computer and chi-square test, which is appropriate to examine potential links between satisfaction items and demographic variables of gender and profession. The correlation coefficient (rho) in the Spearman test was used to identify the strength and direction of the correlation. A chi-square test was also used to compare responses between nurses and physicians. P-values < 0.05 were considered statistically significant.

## Results

A total of 438 completed questionnaires were included in the statistical analysis; 240 of them were physicians and 198 were nurses. One hundred seventy-two (39.3%) respondents were aged 35 years or younger, with 137 (31.3%) between 36 and 45, 102 (23.2%) between 46 and 55 and just 6.2% aged 56 and over. A total of 213 (52.7%) respondents were female, and 207 (47.3%) were male. Four hundred twenty-four (96.8%) respondents had one or more years of experience using computers, and a majority (304, 69.4%) had one or more years of experience using EHR systems. One-hundred ninety-eight (45.2%) respondents were nurses, and 240 (54.8%) were physicians (83 (19%) residents, 99 (22.6%) specialists and 58 (13.2%) consultants). A further breakdown of the physicians' specializations is presented in Table 1.

**Table 1:** Participants' characteristics

Age	N (%)
<=35	172 (39.3%)
36-45	137 (31.3%)
46-55	102 (23.2%)
>55	27 (6.2%)
Total	438 (100%)
Gender	
Male	207 (47.3%)
Female	231 (52.7%)
Total	438 (100%)
Experience using computer	
<1 year	14 (3.2%)
1-3 years	40 (9.1%)
>3 years	384 (87.7)
Total	438 (100%)
Experience using EHR systems	
<1 year	134 (30.6%)
1-3 years	163 (37.2%)
>3 years	141 (32.2%)
Total	438 (100%)
Profession	
Resident doctor	83 (19%)
Specialist doctor	99 (22.6%)
Consultant doctor	58 (13.2%)
Nurse	198 (45.2%)
Physicians specialists	
General doctor	52 (21.7%)
Family doctor	53 (22.1%)
Paediatrician	40 (16.7%)
Surgeon	19 (5.6%)
Psychiatrist	24 (7.9%)
Internal medicine	38 (15.8%)
Others	14 (5.8%)
Total	240

Overall satisfaction

Overall, both physicians and nurses were satisfied with the EHR system (214 (89.5%) vs. 147 (87.9%), p > 0.05), and the majority favoured this system over the previous non-EHR method of information management. A large majority of physicians and nurses (209 (87.1%) vs. 167 (84.3%), p > 0.05) felt that the transition to this new system had been conducted satisfactorily (see Table 2).

**Table 2:** Physicians' and nurses' responses

	Agree responses		Disagree responses		P value
	Physicians N (%)	Nurses N(%)	Physicians N (%)	Nurses N (%)	
Overall satisfaction					
I overall prefer using the system than the old way of doing things.	214 (89.5%)	174 (87.9)	26 (10.8%)	24 (12.12)	0.79
Overall, the introduction of the system has been effective.	209 (87.1%)	167 (84.3%)	31 (12.9%)	31 (15.7%)	0.50
EHR usefulness and usability					
I can depend on the accuracy of the system.	217 (90.4%)	102 (51.5%)	23 (9.6%)	96 (48.5%)	0.001^**^
The use of the system reduces errors.	214 (89.2%)	115 (58.1%)	26 (10.8%)	83 (41.9%)	0.001^**^
I feel the use of system has improved the quality of patient care.	202 (84.2%)	174 (87.9%)	38 (15.8%)	24 (12.1%)	0.205
The system has added to my workload.	115 (47.9%)	133 (67.2%)	125 (52.1%)	65 (32.8%)	0.001^**^
Using the system takes a lot more time than the old way of doing things.	112 (46.7%)	127 (64.1%)	128 (53.3)	71 (35.9%)	0.001^**^
The system facilitates communication of patient information among members of our healthcare team.	168 (70%)	137 (69.2%)	72 (7.9%)	61 (30.8%)	0.95
Members of other disciplines should receive more training regarding how their entry of information affects my use of the system.	176 (73.3%)	129 (65.2%)	64 (26.7)	69 (34.8%)	0.081
EHR training, ongoing support, teamwork and leadership					
The training I received was adequate.	178 (74.2%)	142 (71.7%)	129 (65.2%)	56 (28.3%)	0.65
Adequate resources were available when I was learning to use the system.	174 (72.5%)	141 (71.2%)	66 (27.5%)	57 (28.8%)	0.86
When the system is unavailable, the backup way of doing things works adequately.	165 (68.8%)	97 (49%)	75 (31.2%)	101 (51%)	0.01^*^
I don’t get as much help as I need to fix problems with the system.	168 (77.5%)	153 (77.3)	54 (22.5%)	45 (22.7%)	0.07
People who I work with on a daily basis support me in my use of the system.	222 (92.5%)	183 (92.4%)	18 (7.5%)	15 (7.6%)	0.86
The system facilitates communication of patient information among members of our healthcare team.	178 (73%)	146 (73.7%)	62 (27%)	52 (26.3%)	0.99
People who use the system should have had more to say about the design of the system.	214 (89.2%)	167 (84.3%)	26 (10.8%)	31 (15.7%)	0.18

EHR usefulness and usability

As shown in Table 2, the physicians reported more positive opinions than nurses regarding the dependability of the system’s accuracy (217 (90.4%) vs. 102 (51.5%), p < 0.05) and reported that the EHR system reduced errors (214 (89.2%) vs. 115 (58.1%), p < 0.01), whereas nurses expressed more negative judgements concerning the increased workload and time required to use the new system compared to the previous method (115 (47.9%) vs. 133 (67.2%), p ≤ 0.01 and 112 (46.7%) vs. 127 (64.1%), p ≤ 0.05, respectively). Both physicians and nurses felt that the system improved the quality of care for patients (202 (84.2%) vs. 174 (87.9%), p > 0.05) and improved communication of information between healthcare workers (168 (70%) vs. 137 (69.2%), p > 0.05). However, some respondents (176 (73.3%) physicians vs. 129 (65.2%) nurses, p > 0.05) felt that their colleagues from other disciplines required more training in data entry.

EHR training, ongoing support, teamwork and leadership

Eight of the questionnaire items assessed the adequacy of training and support for the new system. The majority felt that the training had been sufficient (178( 74.2%) physicians vs. 142 (71.7%) nurses, p > 0.05) and that training resources were adequate (174 (72.5%) vs. 141 (71.2%), p > 0.05). However, nurses expressed lower levels of satisfaction with the adequacy of backup systems during downtime and outages (97 (49%) vs. 165 (68.8%), p < 0.01). In terms of clinicians’ satisfaction with teamwork and leadership, most physicians and nurses reported that their colleagues assisted them in using the EHR system (222 (92.5%) vs. 183 (92.4%)), and a majority felt that the system did not interfere with teamwork. However, most physicians and nurses (214 (89.2%) vs. 167 (84.3%)) reported that EHR users should be involved in designing EHR systems.

Demographic attributes, ease of use and satisfaction

Of the demographic variables, age was negatively and significantly correlated with the two satisfaction variables (p < 0.001, rho = -0.18; p < 0.003, rho = -0.14). This result indicates that as age increases, there is a decrease in the overall satisfaction of physicians and nurses, and vice versa. The profession and satisfaction variables also showed a significant positive correlation (p = 0.004, rho = 0.17; p ≤ 0.001, rho = 0.20). However, all of these correlations were weak. The post-hoc test showed that specialist doctors had the highest percentage of overall satisfaction (97/99; 98%). No correlations were found between the satisfaction variables and the remaining demographic variables of gender (p = 0.33), EHR experience (p = 0.48) and computer experience (p = 0.442).

## Discussion

Main findings

This study examined clinicians’ satisfaction levels with an EHR system in a real-world setting in Saudi Arabia and gathered data on these users' perceptions of the factors surrounding EHR implementation. The study found high levels of satisfaction with EHR among the clinicians surveyed, and the majority reported finding the system useful. These findings are in line with those of previous research [1,4,14].

Clinicians’ satisfaction can be influenced by demographic factors. The current study found weak significant correlations between satisfaction and age and satisfaction and profession. The current study showed that there is no significant correlation between overall satisfaction and gender, EHR experience or computer experience. These findings are consistent with previous findings reported by Brown et al. who examined levels of anxiety around computer use among interns and found that gender and computer skill were uncorrelated with satisfaction levels [9]. The lack of correlation between computer experience and satisfaction with EHR is a similar finding to that reported by [13-15]. The lack of correlation between these variables could be due to the provision of sufficient training and support, which have been found to mitigate difficulties that could be experienced by nurses and physicians with no previous experience with technology [8,16]. Alessa et al. stated that adequate training can help overcome usability issues and enhance end-user acceptance [17]. A study by Biruk et al. found that health workers who received adequate training were more than twice as likely to accept the advantages of EHR technology, compared to those whose training was of poor quality [18]. Notably, most participants in the present study (74.2% of physicians and 71.7% of nurses) reported high satisfaction with the EHR training and resources, in contrast to some prior research in which these were reported to be insufficient [4,19].

The association between greater age and lower satisfaction with EHR is also in line with some previous research, which suggests that clinicians who are older are more likely to be dissatisfied with EHR systems, whereas younger clinicians are likely to have higher digital competence and a greater willingness to engage with technology [14,20,21]. However, other research has found these factors to be unrelated [4]. It is important to consider all end users' opinions during the development of healthcare technologies [17], a key consideration that is underscored in the present study, where participants felt that those who use the system should have had more to say about its design. The association between age and satisfaction level suggests that the involvement of end users in the development and implementation of these systems may be particularly important in the case of older users [22]. However, it is also important to note that this issue may decrease as future generations of clinicians are raised with greater access to technology. 

Given the lack of previous research examining both physicians' and nurses’ satisfaction with EHR systems in KSA or the wider Gulf Region, one key finding of the current study was the difference in satisfaction levels between physicians and nurses [1,4,8,9]. Specifically, nurses expressed more dissatisfaction with the adequacy of backup systems during downtime and outages, which can be caused by planned maintenance or unscheduled technical issues. This may be because system outages can create a significant additional workload, including the need to keep both EHR and paper records, with the additional workload falling largely on nurses, negatively affecting their satisfaction [23,24]. This highlights the importance of effective contingency plans to ensure the safety and efficiency of patient care during periods of downtime.

The usefulness of EHRs is a crucial factor that enhances EHR systems’ success. These results indicate high clinician satisfaction with the EHR system’s usefulness, considered an essential factor in influencing clinicians’ attitudes. This is consistent with previous research [1,5,25,26]. The present study also found physicians to have higher satisfaction than nurses regarding the accuracy of the EHR system. Clinicians were also more likely to report that the EHR might reduce medical errors, a finding in line with previous research, helping to ensure patient safety and improve health outcomes [21,27-29]. One possible explanation for this is that physicians noticed this benefit because they have the main responsibility for patients’ treatments in Saudi, meaning doctors are the most actively involved in patients’ treatments and medication-related decisions and ordering. Nurses, on the other hand, were more likely to report that the EHR increased demands on their workload and time, which again may be due to their greater responsibility for managing patient records [26].

The results show that EHRs have the potential to enhance communication of information between healthcare workers, in keeping with previous research [1,21,27,30]. The results also indicate that good data-entry practices among EHR users are considered essential to the system’s success. This finding underscores the importance of improving clinicians’ knowledge of the problems created by inaccurate data entry, which have the potential to cause frustration among fellow users, and reduced quality of care for patients.

Physicians reported that the EHR system had enhanced their work practice, in line with previous research showing positive impacts of EHRs on productivity [4,21,31]. Previous research indicates that clinicians report that EHRs reduce workload [26,32], but other findings contradict this view [26,33-35]. The present study supports the view that EHRs increase workload more among nurses, with satisfaction scores being lowest when they were asked to report on workload and stress associated with EHR use. These issues can be addressed by implementing interfaces that are simple and easy to use and by consulting clinicians during the EHR design phase so as to better meet all end users’ needs and increase their acceptance of the new system during the transition period [26,36].

Previous research indicates that time-saving is a key benefit of EHR systems, helping reduce waiting times for results and records [35,37]. The present study contradicts this finding, with 63.3% reporting that this system took longer to use, a result in line with some other studies [21,38,39]. This may be due to respondents’ relative inexperience with EHRs: 30.8% had less than one year experience with such systems. Previous research reports a decrease in the time required to use EHRs as physicians become more accustomed to them [1,37].

The results reveal high satisfaction with regard to teamwork and leadership. This is in line with previous research, which indicates that these factors have a positive impact on user satisfaction 7 and can improve cooperation among clinicians 1. However, respondents (89.2% of physicians and 84.3% of nurses) reported that clinicians should be consulted during the design of EHRs. This is in keeping with previous studies, which found that the development of user interfaces should incorporate users’ attitudes [1,6,17], that user involvement in design correlates with healthcare professionals’ perceptions [1,40,41] and that such involvement can improve enthusiasm for new systems by increasing clinicians’ sense of ownership [34,37,42,43].

Strengths and limitation

A key strength of this research is its contribution to the developing knowledge regarding clinician satisfaction with EHRs, with implications for numerous stakeholders, including the Ministry of Health (MOH) and hospital managers. Given the specific study context, generalisability beyond Saudi Arabia may be limited but could extend to other cultures and settings similar to Saudi Arabia and the Saudi MOH. A potential limitation of the research is its focus on the opinions of doctors and nurses only; other healthcare professionals or experts might have provided different insights. The use of convenience sampling may also have limited or biased the results obtained. To mitigate this issue, the sample of physicians and nurses was diversified to encompass doctors from multiple departments and with varying levels of education, all of whom utilize EHR, at the premier hospital in Saudi Arabia boasting 700 beds. The small number of participants surveyed and the single location investigated may further impact the generalizability of the study findings. However, these findings should be generalizable to contexts with similar EHR systems and care centres that are similar to the study setting. Finally, the questionnaire used is relatively lengthy, and the time required to complete it could have significantly affected the response rate.

Recommendations for further studies

Based on the current findings, it is important for future studies to investigate the opinions of clinicians and other healthcare professionals using open-ended questions or other qualitative tools (e.g. interviews) to provide in-depth insights into EHR systems and factors that might impact the successful implementation of EHR. There is also a need for research utilizing large-scale study designs to assess EHR systems’ impact on clinicians’ workloads, patient health outcomes and care quality and compare these results with usual care or with different EHR systems implemented in other hospitals, so as to draw clinical conclusions. It is important also to consider patients' perspectives and experiences with EHR systems that could provide valuable insights into the user experience. Finally, the focus of the current study was on clinicians’ satisfaction with EHR; future research should examine the features that clinicians find most useful and compare the usefulness of analogue and EHR systems.

## Conclusions

The research demonstrates that clinicians were mostly satisfied with the EHR system, finding it effective and useful to support their duties, but there were some areas of dissatisfaction among respondents. The physicians reported more positive opinions than the nurses regarding the dependability of the system's accuracy. However, the nurses expressed more dissatisfaction with the EHR's impact on their stress levels and workload, as well as the adequacy of backup systems during downtime and outages. Future large-scale experimental research is recommended to assess the impacts of EHR systems on clinicians’ workload, patient health outcomes and care quality.

## Figures and Tables

**Figure 1:**
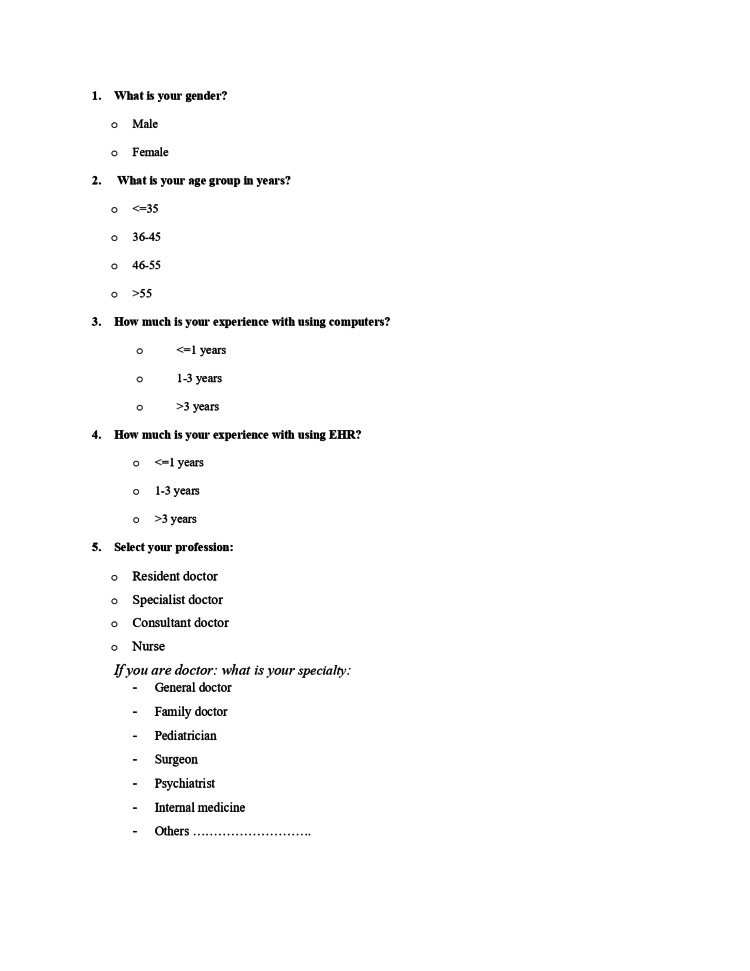
Study tool

## Appendices

Study tool

## References

[1] AlSadrah SA (2020). Electronic medical records and health care promotion in Saudi Arabia: an overview. Saudi Med J.

[2] Bowman S (2013). Impact of electronic health record systems on information integrity: quality and safety implications. Perspect Health Inf Manag.

[3] Lorenzi NM, Kouroubali A, Detmer DE, Bloomrosen M (2009). How to successfully select and implement electronic health records (EHR) in small ambulatory practice settings. BMC Med Inform Decis Mak.

[4] Al-Harthi AAS, Soetanto R, Edum-Fotwe FT (2014). Revisiting client roles and capabilities in construction procurement. Int Conf Constr a Chang World - CIB W92 Procure Syst Sri Lanka.

[5] Glovers Glovers, Lucinda; Woods, M. (2020 (2020). https://www.commonwealthfund.org/international-health-policy-center/countries/australia.

[6] Abdullah Alharbi R (2023). Adoption of electronic health records in Saudi Arabia hospitals: knowledge and usage. J King Saud Univ Sci.

[7] Jabali AK, Abdulla FA (2023). Electronic health records perception among three healthcare providers specialties in Saudi Arabia: a cross-sectional study. Healthc Technol Lett.

[8] Jabali A (2021). Predictors of anesthesiologists' attitude toward EHRs in Saudi Arabia for clinical practice. Inform Med Unlocked.

[9] Brown SH, Coney RD (1994). Changes in physicians' computer anxiety and attitudes related to clinical information system use. J Am Med Inform Assoc.

[10] Logeswaran A, Chong YJ, Edmunds MR (2020). The electronic health record in ophthalmology: usability evaluation tools for health care professionals. Ophthalmol Ther.

[11] McBride S, Tietze M, DeSalvo K, McBride S, Tietze M, Bickford CJ (2016). Nursing informatics for the advanced practice nurse 2nd ed. Patient safety, quality, outcomes, and interprofessionalism. Nursing Informatics for the Advanced Practice Nurse [Internet]. 2nd ed. Patient Safety, Quality, Outcomes, and Interprofessionalism. New York: Springer Publishing Company.

[12] Gugerty B, Maranda M, Rook D (2006). The clinical information system implementation evaluation scale. Stud Health Technol Inform.

[13] Lozano LA, Barthold M, Judkins-Cohn T (2014). Using the clinical information system implementation evaluation scale as a clinical implementation strategy. Comput Inform Nurs.

[14] Alasmary M, El Metwally A, Househ M (2014). The association between computer literacy and training on clinical productivity and user satisfaction in using the electronic medical record in Saudi Arabia. J Med Syst.

[15] Penrod LE, Gadd CS (2001). Attitudes of academic-based and community-based physicians regarding EMR use during outpatient encounters. Proc AMIA Symp.

[16] Al Alawi S, Al Dhaheri A, Al Baloushi D, Al Dhaheri M, Prinsloo EA (2014). Physician user satisfaction with an electronic medical records system in primary healthcare centres in Al Ain: a qualitative study. BMJ Open.

[17] Alessa T, S Hawley M, Alsulamy N, de Witte L (2021). Using a commercially available app for the self-management of hypertension: acceptance and usability study in Saudi Arabia. JMIR Mhealth Uhealth.

[18] Biruk S, Yilma T, Andualem M, Tilahun B (2014). Health Professionals' readiness to implement electronic medical record system at three hospitals in Ethiopia: a cross sectional study. BMC Med Inform Decis Mak.

[19] Tilahun B, Fritz F (2015). Modeling antecedents of electronic medical record system implementation success in low-resource setting hospitals. BMC Med Inform Decis Mak.

[20] Sykes TA, Venkatesh V, Rai A (2011). Explaining physicians' use of EMR systems and performance in the shakedown phase. J Am Med Inform Assoc.

[21] Al Otaybi HF, Al-Raddadi RM, Bakhamees FH (2022). Performance, barriers, and satisfaction of healthcare workers toward electronic medical records in Saudi Arabia: a national multicenter study. Cureus.

[22] Marcilly R, Lesselroth B, Guerlinger S, Pigot A, Schiro J, Pelayo S (2023). Active involvement of end-users in an EHR procurement process: a usability walkthrough feasibility case study. J Gen Intern Med.

[23] Larsen E, Hoffman D, Rivera C, Kleiner BM, Wernz C, Ratwani RM (2019). Continuing patient care during electronic health record downtime. Appl Clin Inform.

[24] Kaipio J, Kuusisto A, Hyppönen H, Heponiemi T, Lääveri T (2020). Physicians' and nurses' experiences on EHR usability: Comparison between the professional groups by employment sector and system brand. Int J Med Inform.

[25] Hsieh PJ (2015). Physicians' acceptance of electronic medical records exchange: an extension of the decomposed TPB model with institutional trust and perceived risk. Int J Med Inform.

[26] Abdulah D, Perot K (2022). Barriers and benefits of adopting electronic health records (EHRs) in public hospitals. Heal Probl Civiliz.

[27] McGuire MJ, Noronha G, Samal L, Yeh HC, Crocetti S, Kravet S (2013). Patient safety perceptions of primary care providers after implementation of an electronic medical record system. J Gen Intern Med.

[28] Williams DC, Warren RW, Ebeling M, Andrews AL, Teufel Ii RJ (2019). Physician use of electronic health records: survey study assessing factors associated with provider reported satisfaction and perceived patient impact. JMIR Med Inform.

[29] Nguyen OT, Jenkins NJ, Khanna N, Shah S, Gartland AJ, Turner K, Merlo LJ (2021). A systematic review of contributing factors of and solutions to electronic health record-related impacts on physician well-being. J Am Med Inform Assoc.

[30] Kirkendall ES, Goldenhar LM, Simon JL, Wheeler DS, Andrew Spooner S (2013). Transitioning from a computerized provider order entry and paper documentation system to an electronic health record: expectations and experiences of hospital staff. Int J Med Inform.

[31] Omboni S, Padwal RS, Alessa T (2022). The worldwide impact of telemedicine during COVID-19: current evidence and recommendations for the future. Connect Health.

[32] Taft T, Lenert L, Sakaguchi F, Stoddard G, Milne C (2015). Effects of electronic health record use on the exam room communication skills of resident physicians: a randomized within-subjects study. J Am Med Inform Assoc.

[33] Kemper AR, Uren RL, Clark SJ (2006). Adoption of electronic health records in primary care pediatric practices. Pediatrics.

[34] Simon SR, Kaushal R, Cleary PD (2007). Physicians and electronic health records: a statewide survey. Arch Intern Med.

[35] Martín-Méndez ME, García-Díaz V, Zurrón-Madera P, Fernández-Feito A, Jimeno-Demuth F, Lana A (2021). Evolution of nursing workload indicators since the implementation of the electronic health record at a tertiary hospital in Spain. Comput Inform Nurs.

[36] Alanazi B, Butler-Henderson K, Alanazi M (2020). Perceptions of healthcare professionals about the adoption and use of EHR in Gulf Cooperation Council countries: a systematic review. BMJ Health Care Inform.

[37] Cucciniello M, Lapsley I, Nasi G, Pagliari C (2015). Understanding key factors affecting electronic medical record implementation: a sociotechnical approach. BMC Health Serv Res.

[38] Vishwanath A, Singh SR, Winkelstein P (2010). The impact of electronic medical record systems on outpatient workflows: a longitudinal evaluation of its workflow effects. Int J Med Inform.

[39] Calder-Sprackman S, Clapham G, Kandiah T (2021). The impact of adoption of an electronic health record on emergency physician work: A time motion study. J Am Coll Emerg Physicians Open.

[40] Castillo VH, Martínez-García AI, Pulido JR (2010). A knowledge-based taxonomy of critical factors for adopting electronic health record systems by physicians: a systematic literature review. BMC Med Inform Decis Mak.

[41] Alessa T, Hawley MS, Hock ES, de Witte L (2019). Smartphone apps to support self-management of hypertension: review and content analysis. JMIR Mhealth Uhealth.

[42] Sipanoun P, Oulton K, Gibson F, Wray J (2022). The experiences and perceptions of users of an electronic patient record system in a pediatric hospital setting: a systematic review. Int J Med Inform.

[43] Alhur A (2024). Overcoming electronic medical records adoption challenges in Saudi Arabia. Cureus.

